# Assessment of the Weldability of T-Welded Joints in 10 mm Thick TMCP Steel Using Laser Beam

**DOI:** 10.3390/ma11071192

**Published:** 2018-07-11

**Authors:** Jacek Górka

**Affiliations:** Department of Welding Engineering, Silesian University of Technology, Konarskiego 18A, 44-100 Gliwice, Poland; jacek.gorka@polsl.pl; Tel.: +48-32-237-1445; Fax: +48-32-237-1232

**Keywords:** laser beam, T-joints, weldability, Thermomechanically Controlled Processed, TMCP steel

## Abstract

The article presents tests aimed to verify the possibility of Thermomechanically Controlled Processed (TMCP) steels T-joints laser welding. The 10 mm thick high-yield-point steel S700MC obtained in an industrial manufacturing process was used for tests of laser welding. The joints made during the tests were single- and double-sided. Subsequent nondestructive tests revealed that the laser-welded joints represented quality level B in accordance with PN-EN ISO 13919-1. Single-sided welding performed at the output laser beam power of 11 kW provided the penetration depth of just 8 mm without visibly deforming of the joint. The double-sided welded joints were characterized by proper geometry and the presence of gas pores in the welds not compromising the requirements of quality level B (strict requirements). The identified weld structure was bainitic-ferritic. The weld hardness was by approximately 60 HV1 higher than that of the base material (280 HV1). The HAZ (Heat Affected Zone) area was slightly softer than the base material. The tests of thin foils performed using a high-resolution scanning transmission electron microscope revealed that, during welding, an increase in the content of the base material in the weld was accompanied by an increase in contents of alloying microagents Ti and Nb, particularly near the fusion line. The above-named alloying microagents, in the form of fine-dispersive (Ti,Nb)(C,N) type precipitates, could reduce plastic properties of joints.

## 1. Introduction

Laser welding is one of many technologies enabling the joining of materials. Because of its unquestionable advantages, the above-named technology has proved successful when joining small-sized and simply-shaped elements. However, presently, technological progress combined with increased laser power enable the making of joints in areas previously almost entirely dependent on conventional welding techniques [1,2,3]. The increasingly fast replacement of long-used welding techniques by modern technologies is dictated by the use of new high-strength materials, the joining of which requires precise techniques and appropriate technologies, without compromising high mechanical properties of the base material. Laser welding, in addition to its high precision (previously quite paradoxically regarded as a disadvantage because of very precise and thus expensive preparations required prior to welding), enables the very fast welding of elements without the use of filler metals while not affecting the properties of the base material [4,5,6,7]. Technical and economic aspects resulting from the possibility of using high-yield-point Thermomechanically Controlled Processed (TMCP) steels in energy-saving integrated production lines and the usability of the above-named steels when making various structures, including those exposed to extremely harsh climate, are responsible for the growing scientific interest in this group of materials and improvements in their manufacturing and joining (e.g., using the laser beam) [8,9,10,11,12]. Most research works related to the weldability of TMCP steels are concerned with materials having a yield point of up to 460 MPa. According to the aforementioned works, because of lower contents of alloying constituents (particularly carbon), TMCP steels are characterised by significantly lower susceptibility to hardening in the HAZ than normalised steels. The HAZ of TMCP steels contains a narrow area softer than the base material and characterised by slightly lower mechanical properties [13,14,15,16]. The authors state that the narrow width of the above-named area does not significantly affect mechanical properties of welded joints. However, it appears that, particularly in terms of high-yield-point steels, the above-named area could undergo sensitisation to brittle and fatigue cracking and become characterised by worsened plastic properties [8,9,10,11,12,13,14,15,16,17]. Initially, the structure of TMCP steels is fine-grained and, in addition, precipitation- and strain-hardened. Rolling parameters such as temperature, strain degree, and cooling rate are adjusted to the kinetics of the precipitation of intermetallic phases of microagents in steels. The presence of vanadium, titanium, and niobium in the base material affects positively the formation of the fine-grained structure in the weld metal and HAZ [18,19,20,21]. This is because of the presence of fine precipitates of titanium nitrides as well as vanadium and niobium carbonitrides, restraining the growth of austenite grains. Welding can disturb the course of the above-named processes, leading to the uncontrolled precipitation of MX-type phases. In addition, an excessively high nitrogen content in the base material accompanied by insufficient contents of free nitrogen-bonding elements (i.e., aluminium and, particularly, titanium) can result in ageing, deteriorating the weldability of steels [22,23,24]. Until recently, laser welding has rarely seen its industrial implementations, primarily because of high investment costs and technological difficulties. Presently, laser technologies no longer pose high-risk investments as was the case only a few years ago. In addition, the future market competition is most likely to involve companies extensively utilising welding technologies as increasingly many welding engineering sectors are interested in industrial applications of technological lasers [25,26,27]. The use of technological lasers in production processes imposes the modernisation of such processes and makes it possible to produce technologically advanced new generations of products. Increasingly often, laser welding finds its application in the making of T-joints of thick sheets or plates (i.e., thicker than 4 mm). Presently, laser welding is used not only in the automotive industry but also in the shipbuilding and power engineering industrial sectors, where the making of thick T-joints is often required. The application of laser welding enables high-quality T-joints to be obtained without the necessity of using filler metals, thus significantly lowering production costs and reducing welding strains in the aforesaid types of joints [28,29,30].

## 2. Experimental Section

**Figure 1:**
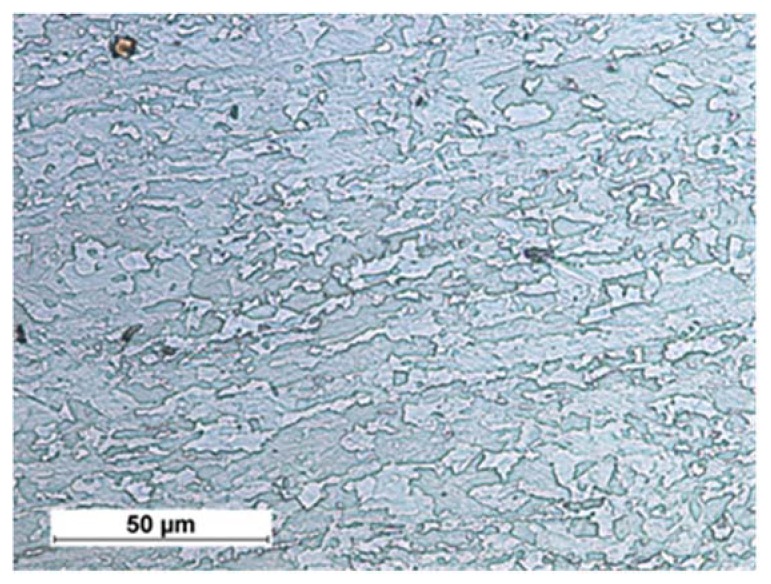
Structure of bainitic-ferritic steel S700MC with visible effects of plastic deformation.

The tests aimed to verify the possibility of making welded joints in TMCP steels using the laser beam without the filler metal. The tests involved 10 mm thick, high-yield-point steel S700MC obtained in the industrial manufacturing process. The chemical composition and properties of the steel are presented in Table 1, whereas the structure of the steel is presented in Figure 1.

Steel S700MC is characterised by the nonequilibrium, fine-grained, bainitic-ferritic structure and a relatively low carbon equivalent (0.33%) (i.e., good weldability attributes). High mechanical properties of steel S700MC are obtained through the thermomechanical control process (TMCP) combined with the use of alloying microagents such as Ti and Nb, as well as a small amount of V. The total content of alloying microagents amounts to 0.17% by weight. The above-presented value does not exceed the permissible content of 0.22% in relation to steels with microagents. Steel S700MC is characterised by a very low carbon content (0.056%). The aforesaid content can reduce the effect of austenitic transformations during welding thermal cycles on mechanical and plastic properties of welded joints.

### 2.1. Welding Process

**Figure 2:**
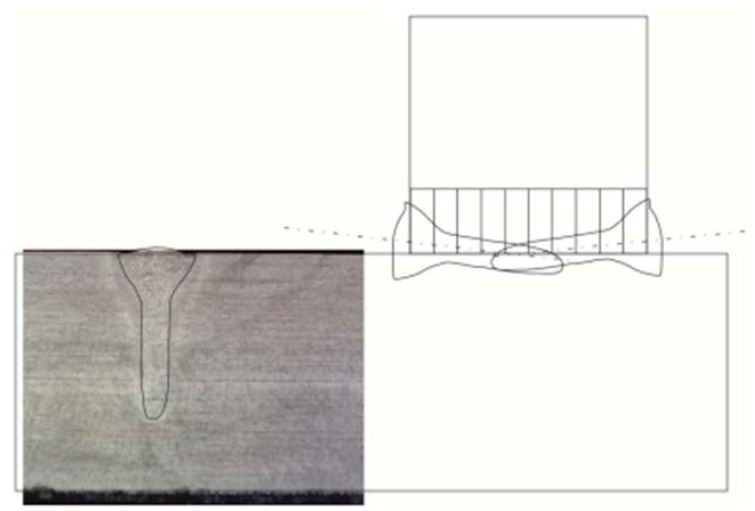
Macrostructure of the plate penetration run performed using a laser beam power of 7 kW, the outlined fusion line, and the outline of the penetration run plotted on the schematic diagram of the T-joint.

To adjust appropriate parameters enabling the obtainment of proper T-joints, it was necessary to perform bead-on-plate welding tests using various process parameters. An adopted bead-on-plate welding speed of 2 m/min was constant, whereas parameters subjected to changes included the power of the laser beam and the position of the laser beam focus. The above-presented bead-on-plate welding speed ensured that the efficiency of the laser welding of T-joints would be significantly higher than that obtained using arc methods when welding the same type of joints. It was noticed that the bead-on-plate welding process was more stable (fewer spatters formed) when the laser beam focus penetrated the material subjected to welding (negative values of parameter f). Metallographic tests were used to determine the geometrical dimensions of the penetration run in relation to a given group of parameters. Afterwards, a graphic software GIMP2.10.4 (Free Software Foundation, Inc., Boston, MA, USA) programme was used to outline the fusion lines of the plate penetration run and to plot the above-named shape of the weld on the schematic diagram of the T-joint (Figure 2). The foregoing combined with theoretical calculations of the focused beam divergence angle made it possible to adjust the power parameters of the double-sided welding of T-joints, an appropriate laser beam insertion angle, and the position of the laser beam focus in relation to the interface of plates to be joined:

1. Laser beam insertion angle: α = 6°
2. Lift-off of the laser beam in relation to the T-joint base: a = 0.5 mm

**Figure 3:**
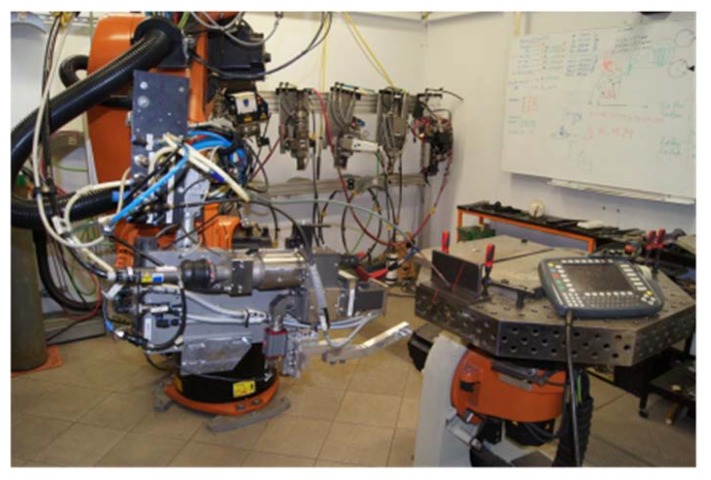
Station for the laser welding of T-joints.

The bead-on-plate welding and joints welding tests were performed using a TruLaser Robot 5120 robotic laser welding station (TRUMPF GmbH + Co. KG, Ditzingen, Germany) (TRUMPF). The station included a TruDisk 12002 disc laser having a maximum power of 12 kW and provided with a working head attached to a six-axis KUKA KR30HA industrial robot (Figure 3). The welding of the T-joints also involved the use of a D70 head (TRUMPF) connected to the disc laser using an optical fibre having a diameter of 0.3 mm. The laser welding head used in the tests was characterised by the following optical parameters:

1. collimator lens focal length: f_col_ = 200 mm
2. focusing lens focal length: f_foc_ = 400 mm

The above-presented optical parameters of the head and the use of the above-named optical fibre enabled the obtainment of the laser beam characterised by the following parameters (theoretical calculations):

1. Laser beam focus diameter d_foc_ = 0.6 (mm)
2. Rayleigh length Z_R_ = 7.5 (mm)
3. Focused laser beam divergence angle Θ_f_ = 4.6 (deg.)
4. Laser beam quality parameter BPP = 12 (mm·mrad)

**Figure 4:**
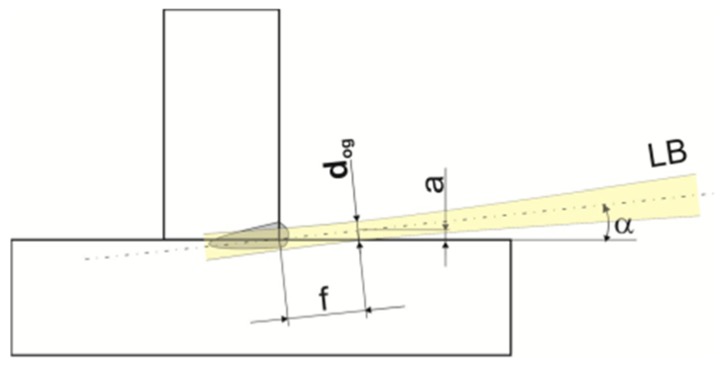
Schematic diagrams of primary geometrical correlations between the position of the laser beam focus having diameter d_foc_ and the interface (line) of the plates in the welded joint area.

The welding tests were performed using various laser beam power values and various laser beam focus positions in relation to the laser beam propagation axis and in relation to the T-joint web surface, that is, parameter f (Figure 4). A negative value of parameter f indicates the penetration of the laser beam focus into a material subjected to welding. In case of double-sided welds, the specimen was turned after the first run and the welding process was continued after the joint had cooled to a temperature of 40°.

**Figure 5:**
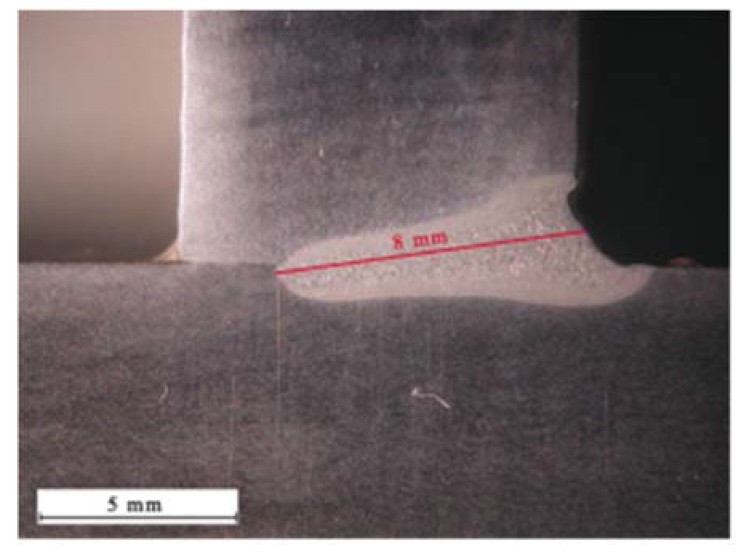
Maximum penetration during single-sided welding.

**Figure 6:**
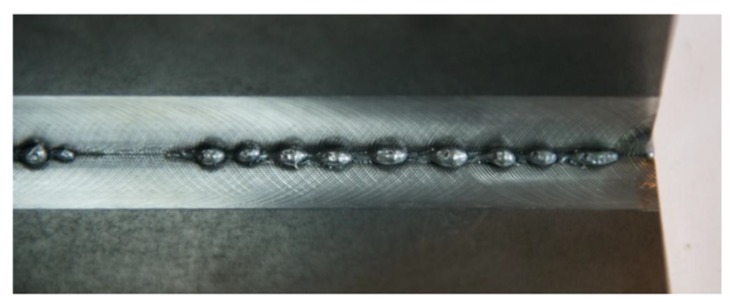
Single-sided T-joint with excessive penetration on the root side.

The initial welding tests revealed that it was difficult to perform the stable single-sided welding of 10 mm thick T-joints. The maximum penetration obtained using stable process parameters amounted to 8 mm (Figure 5). The formation of the weld root proved problematic. The obtainment of full penetration was accompanied by the liquid metal forming local regular excessive penetration on the weld root side (Figure 6).

**Figure 7:**
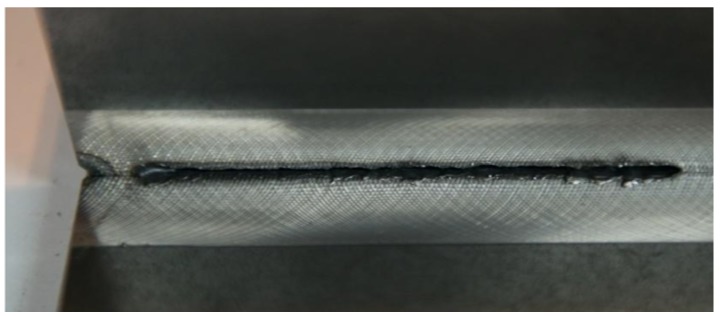
Single-sided T-joint with the incompletely filled groove.

**Figure 8:**
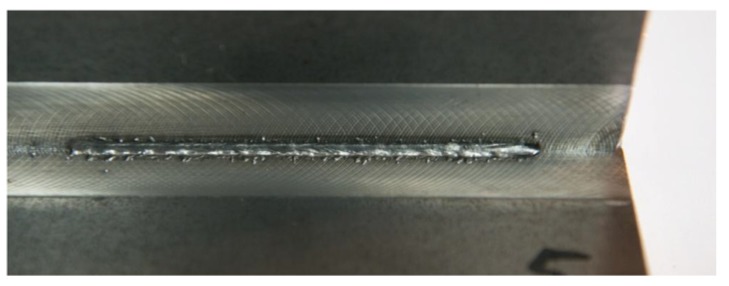
Double-sided T-joint with the proper weld face.

At the same time, on the weld face side, it was possible to observe the incompletely filled groove along the entire length of the joint (Figure 7). Because of the very narrow window of stable single-sided welding parameters, it was ascertained that an alternative (competitive) solution could include double-sided welding, ensuring the formation of the proper weld face and providing significant flexibility as regards changes in welding process parameters (Figure 8). The welding process parameters in relation to selected joints are presented in Table 2.

### 2.2. Tests of Welded Joints

The test welded joints were subjected to visual tests following the requirements of PN-EN ISO 17637:2011 standards [31]. Afterwards, the joints were subjected to the following destructive tests:

1. Macroscopic metallographic tests involving the use of an Olympus SZX9 light stereoscopic microscope (Olympus, Tokyo, Japan); the test specimens were etched using Adler’s reagent (CHMES, Poznań, Poland);
2. Microscopic metallographic tests involving the use of a NIKON ECLIPSE MA100 light microscope (Nicon, Tokyo, Japan); the test specimens were etched using Nital (CHMES, Poznań, Poland);
3. Quantitative tests concerning contents of microagents hardening steel S700MC, performed using a JXA-8230 electron microprobe X-ray analyser (JEOL) (JEOL Ltd., Akishima, Japan) and wavelength dispersion spectroscopy (WDS) (JEOL Ltd., Akishima, Japan);
4. Tests of thin foils using a Titan 80–300 kV high-resolution scanning transmission electron microscope (HR S/TEM) (FEI) (Thermo Fisher Scientific, Gainesville, FL, USA) equipped with an XFEG electron gun featuring Schottky field emission of enhanced brightness;
5. Phase analysis-related X-ray tests (PANalytical, Almelo, The Netherlands) performed using an X’Pert PRO diffractometer and an X’Celerator strip detector;
6. Vickers hardness test performed using a WILSON WOLPERT 430 hardness tester (Wilson Wolpert, Aachen, Germany), following the requirements of PN-EN ISO 9015-1 [32];
7. Assessment of a welding imperfection (gas pores), following the requirements of PN-EN ISO 13919-1:2002 [33];
8. An attempt to bend the T-joint on a ZWICK/ROELL Z 330RED testing machine) (Zwick Roell, Ulm, Germany) (the bending test specimen was cut mechanically from the weld joint; width of the test sample was 45 mm).

## 3. Results and Discussion

**Figure 9:**
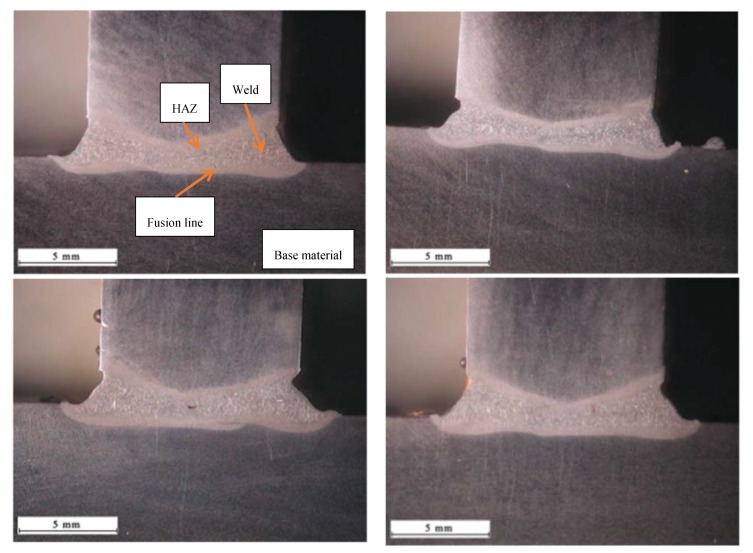

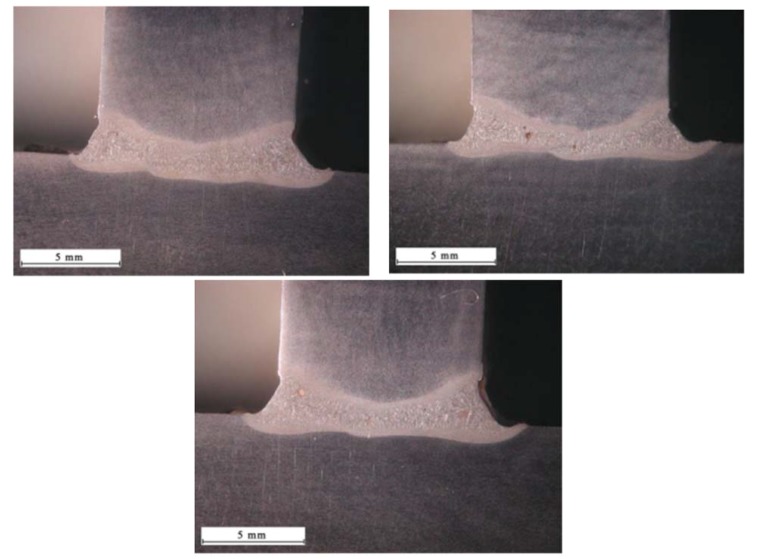
Macrostructure of the T-joint in order 1–7, (Table 2).

The research-related visual tests enabled the elimination of joints characterised by the lack of penetration or excessive penetration on the root side. The remaining, double-sided joints were characterised by the proper geometry of the welds (Figure 9). 

**Figure 10:**
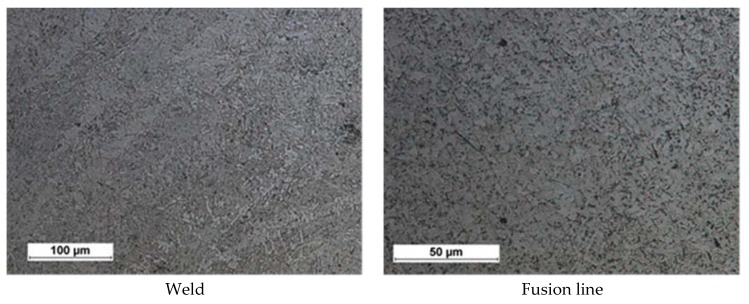

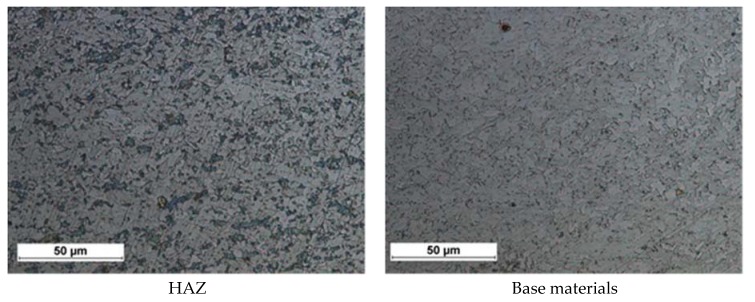
Example microstructure of the welded joint No. 4, right weld of joint.

The microscopic tests concerning the weld area revealed the bainitic-ferritic structure. The HAZ area did not reveal any significant changes in the grain size in relation to the base material. The foregoing was related to a very low heat input to the welded joint area during the laser welding process (Figure 10).

**Figure 11:**
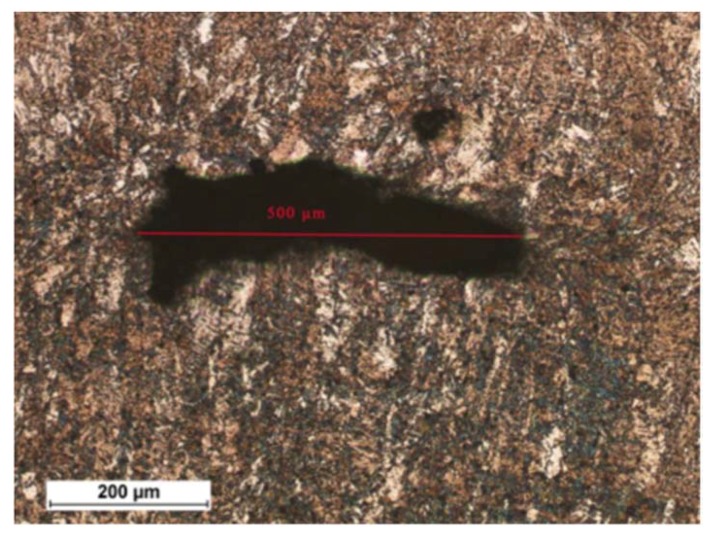
Size of the gas pore in joint No. 3.

The microscopic tests revealed the presence of gas pores in the welded joints. The formation of the above-named gas pores could be ascribed to the fast crystallisation of the liquid metal pool triggered by very high welding rates responsible for the impeded release of gases from the weld zone. The gas pores could form as a result of the confinement of gases dissolved in the metal or as a result of the evaporation of alloying elements or impurities, if any, located on the surface of the interface of the joined sheets. The identification of the significance of the above-named imperfection required the performance of verification tests based on the PN-EN ISO 13919-1:2002 standard [33] (Table 3). The welded joint subjected to the verification of quality (i.e., joint No. 3) was characterised by the presence of the greatest gas pore (Figure 11).

The largest welding imperfection of the T-joints satisfied the requirements of quality level B, therefore the remaining joints also satisfied the requirements of quality level B.

**Figure 12:**
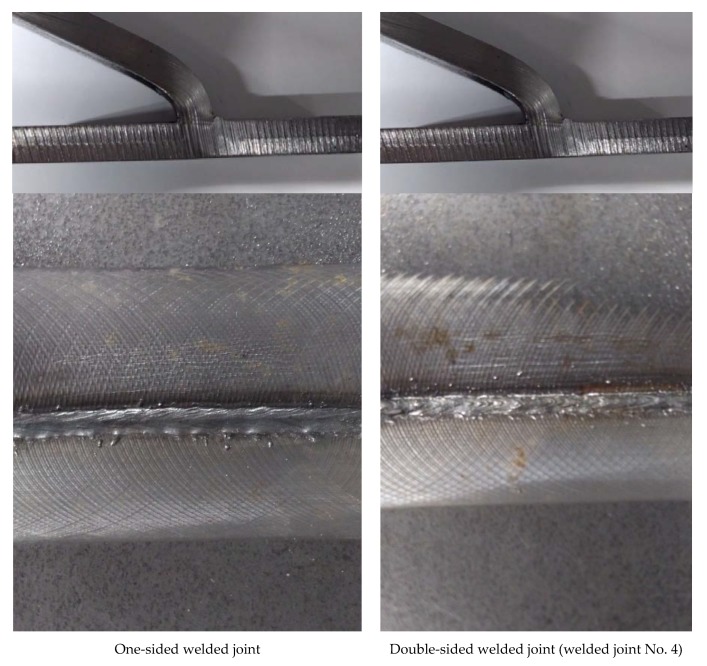
View of joints after bending test.

Bending test of welded joints with one and two sides showed very high strength and plastic properties, Figure 12.

**Figure 13:**
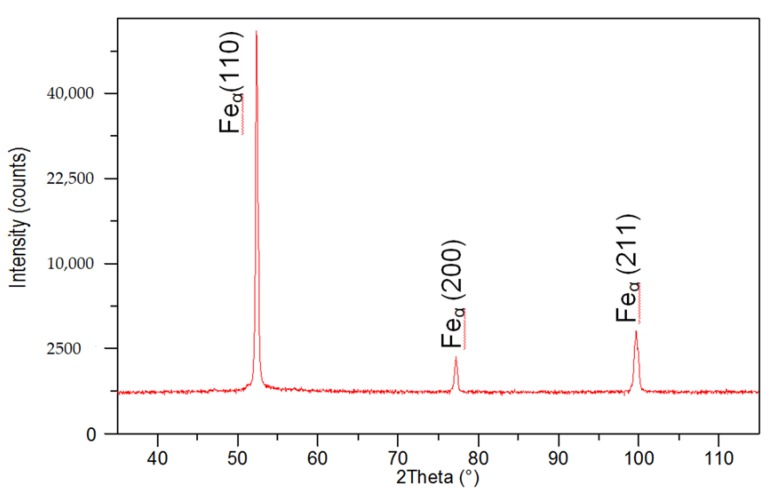
XRD pattern of the weld of the laser-welded joint made of steel S700MC.

The X-ray phase analysis revealed that the base material and the weld made using the laser beam were entirely composed of the ferritic phase (Figure 13).

**Figure 14:**
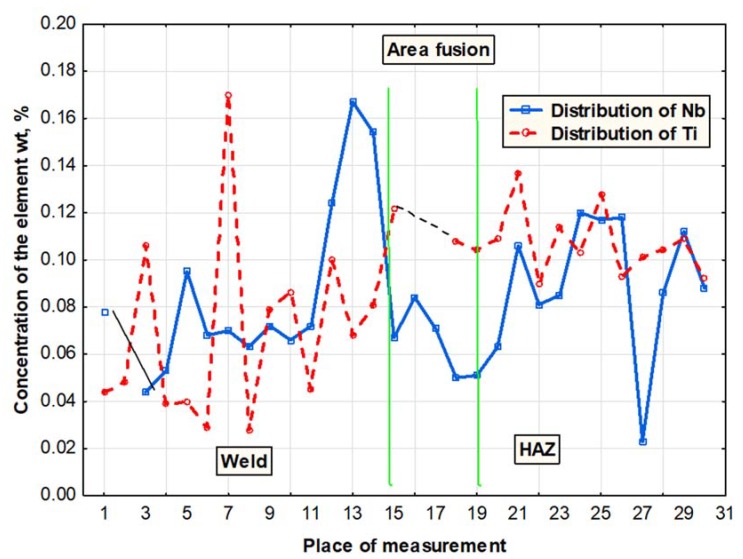
Distribution of Ti and Nb in the fusion area of the laser-welded joint made of steel S700MC.

In the laser welds made without the use of the filler metal, the contents of Ti and Nb were significantly higher than those in the welds made using the filler metal. Higher contents of hardening constituents could lead to decreased toughness in the weld area in comparison with that of the base material. An increase in the contents of microalloy elements was particularly visible near the fusion line, which was confirmed by the detailed analysis of the chemical composition performed using an electron microprobe X-ray analyser (Figure 14). Certain weld areas near the fusion line contained high amounts of titanium and niobium, which indicated the presence of clusters of carbonitride precipitates which had not dissolved entirely in the liquid metal pool. The excessively high concentration of hardening phases in the fusion area could have a highly adverse effect on the plastic properties of the weld [34,35].

**Figure 15:**
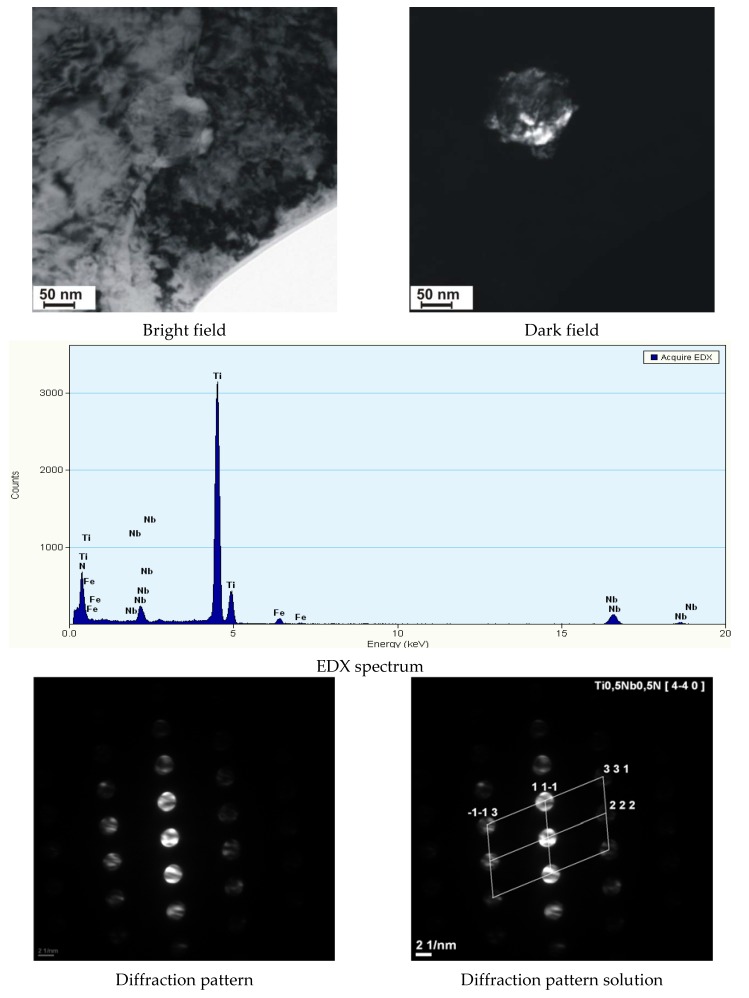
Precipitate of the (Ti,Nb)N nitride along with the fine spherical hardening precipitates in the weld of steel S700MC, made using the laser beam.

The significant content of hardening phases in the weld during cooling led to the significant precipitation hardening through fine-dispersive (Ti,Nb)(C,N) type precipitates (of several nm in size), precipitated near greater (Ti,Nb)N particles (100 nm in size) (Figure 15), which reduced the plastic properties of the welds.

**Figure 16:**
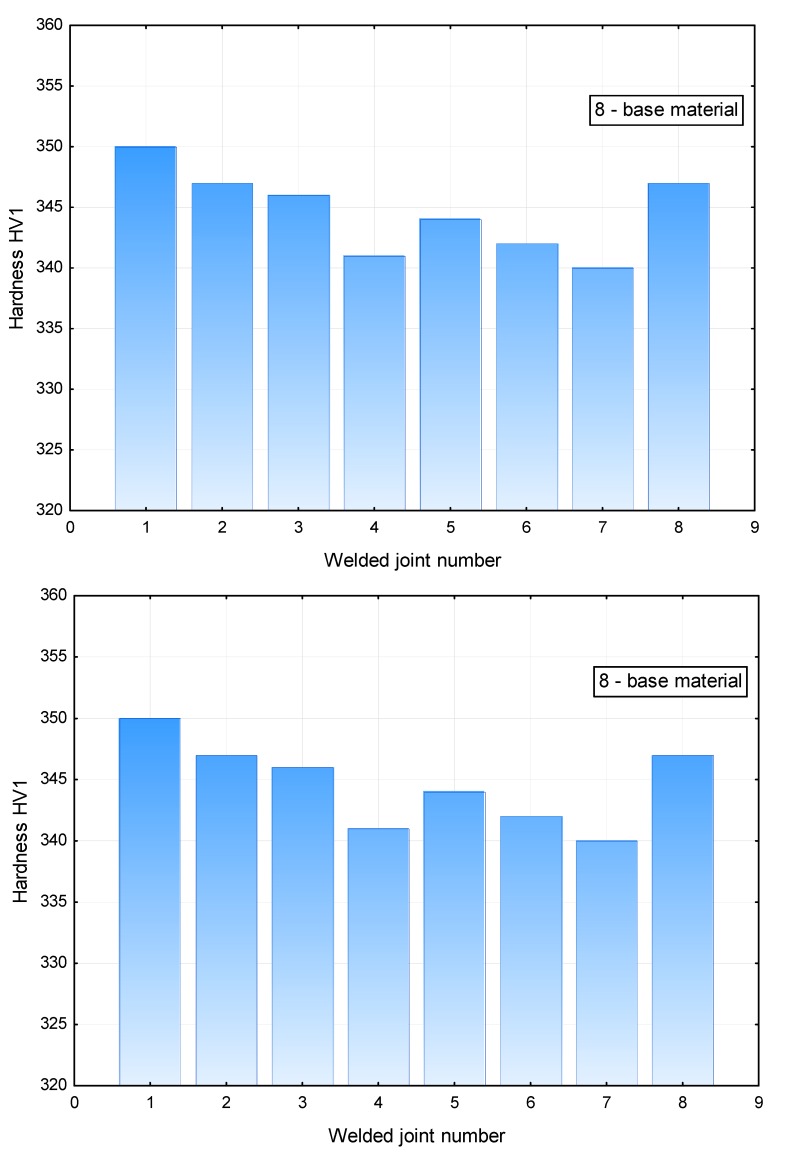
Averaged hardness in the weld area of the T-joints.

**Figure 17:**
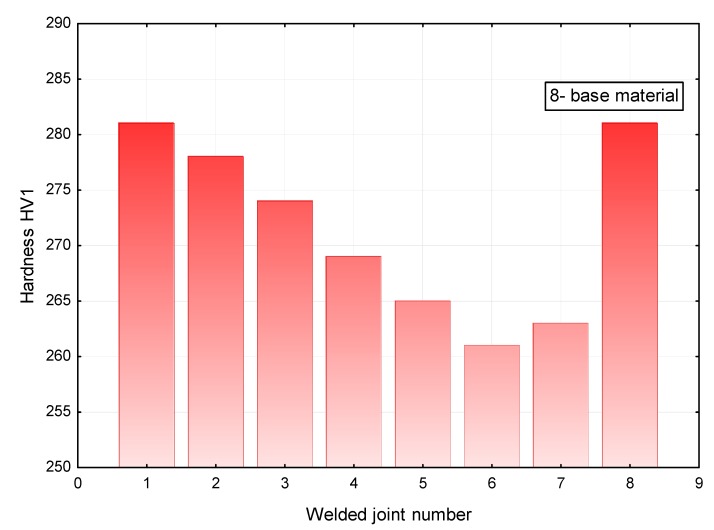
Averaged hardness in the HAZ area of the T-joints.

The analysis of the hardness measurement results revealed that a change in the laser beam power during welding performed at a constant welding rate did not significantly affect the hardness in the weld area. The hardness of the weld was restricted within the range of 340 HV1 to 350 HV1, whereas the hardness of the base material amounted to 280 HV1 (Figure 16). The HAZ area hardness was significantly affected by welding linear energy and the position of the laser beam focus. In terms of the HAZ, the highest hardness was observed in the joints made using a laser beam power of 7 kW (approximately 280 HV1). An increase in the laser beam power to 11 kW was accompanied by a decrease in the hardness of the HAZ area to 260 HV1 (Figure 17). Because of the contact hardening phenomenon, the above-named softening of the HAZ did not reduce the mechanical properties of the welded joint.

## 4. Conclusions

The tests concerning the laser welding of T-joints made in 10 mm thick steel S700MC revealed that the use of the TruDisk 12002 laser having a maximum power of 12 kW enabled the obtainment of high-quality T-joints satisfying the requirements of quality level B in accordance with the PN-EN ISO 13919-1 standard [33] and characterised by very good geometrical shapes of penetration. Because of the minimum angle of the welding head position, it was possible to obtain the 8 mm deep penetration of the plates after the single-sided run of the laser beam. In turn, the double-sided welding process enabled the obtainment of the high-quality butt connection of the T-joint made of 10 mm thick plates (full penetration). Ensuring the quality is very important, because there are a lot of T-joints in welded structures, both with butt welds and fillet welds where cracks can occur and cause structural failures. The obtained T-joints, both single and double-sided, are characterized by their own strength properties, which were confirmed by the bending test. The weld area contained single gas pores, yet their sizes did not reduce the operational properties of the welded joints. The bainitic-ferritic weld structure was characterised by a hardness of approximately 350 HV1. In turn, the HAZ area was characterised by the slight growth of grains and the partial loss of properties previously obtained in the thermomechanical control process. The foregoing resulted in the formation of a zone softer than the base material (260 HV1). The above-named slight softening did not reduce the mechanical properties of the joints. During laser welding performed without the use of the filler metal, the contents of alloying microagents (Ti and Nb) in the weld were the same as those in the base material. The significant content of hardening phases in the weld during cooling resulted in the significant precipitation hardening by fine-dispersive (Ti, Nb)(C, N) type precipitates, which could lead to the deterioration of the plastic properties of the welds. Laser welding is definitely more efficient than classical arc welding methods, so it ensures higher production efficiency. Due to the high energy density of the laser beam, the penetration depths obtained are greater compared to the classical methods. It can be visible in higher toughness properties of the T-joints.

## Figures and Tables

**Table 1:** Chemical composition of steel S700MC 10 mm thick plates.

Contents of Chemical Elements, % by Weight										
C	Mn	Si	S	P	Al	Nb	Ti	V	N *	C_e_ **
0.056	1.68	0.16	0.005	0.01	0.027	0.044	0.12	0.006	72	0.33
Mechanical properties										
Tensile strength R_m_, MPa			Yield point R_e_, MPa			Elongation A_5_, %		Toughness, J/cm^2^ (−20 °C)		
822			768			19		135		

*—N: content in ppm; nitrogen was identified using the high-temperature extraction method *—total content of Nb, V and Ti should amount to a maximum of 0.22%; ** C_e_—carbon equivalent.

**Table 2:** Parameters adjusted during the laser welding of T-joints made of 10 mm thick steel S700MC.

Joint Designation	Laser Beam Power P, kW	Welding Rate v, m/min	Focus Position f, mm	Laser Beam Lift-off a, mm
Joint 1	6	2	−4	0.5
Joint 2	6	2	−6	0.5
Joint 3	7	2	−4	0.5
Joint 4	7	2	−6	0.5
Joint 5	7	2	−8	0.5
Joint 6	8	2	−6	0.5
Joint 7	11	2	−8	0.5

**Table 3:** Ultimate limit sizes of welding imperfections in the form of gas pores in accordance with PN-EN ISO 13919-1:2002 standard [33].

Ultimate Limit Sizes of Welding Imperfections in Relation to Quality Levels		
Lenient requirements D	Medium requirements C	Strict requirements B
l or h ≤ 0.5 t or 5 mm; lower value applies, f ≤ 6%	l or h ≤ 0.4 t or 3 mm; lower value applies, f ≤ 2%	l or h ≤ 0.3 t or 2 mm; lower value applies, f ≤ 0.7%

Key: l—length of a welding imperfection (measured in any direction), h—dimension of a welding imperfection (height, width), t—thickness of an element subjected to welding, f—gas pore or gas cavity projection areas. Calculations: h = 500 μm = 0.5 mm, t = 10 mm. 0.5 mm ≤ 0.3 × 10 mm, 0.5 mm ≤ 3 mm, 0.5 mm ≤ 2 mm. Satisfied requirements of quality level B.
